# A Case of Nonimmune Hydrops Fetalis Caused by Homozygous α-Thalassemia

**DOI:** 10.4274/tjh.2012.0021

**Published:** 2013-03-05

**Authors:** Melek Akar, Dilek Dilli, Uğur Dilmen

**Affiliations:** 1 Zekai Tahir Burak Maternity Teaching Hospital, Department of Neonatology, Ankara, Turkey; 2 Yıldırım Beyazıt University Faculty of Medicine and Director General of Health Research, Ministry of Health, Department of Pediatrics and Neonatology, Ankara, Turkey

**Keywords:** α-Thalassemia, Hydrops fetalis, Nonimmune

## Abstract

Hydrops fetalis is a serious condition which indicates poor prognosis for the affected fetus. Although the incidence of isoimmune hydrops fetalis has decreased markedly, nonimmune hydrops fetalis cases have been more frequently reported. Nonimmune-mediated hydrops can be caused by hemoglobinopathies. In this report we present a case of nonimmune hydrops fetalis caused by homozygous α-thalassemia. Because of the high incidence of the disease in our country, α-thalassemia should be investigated in all cases with nonimmune hydrops fetalis.

**Conflict of interest:**None declared.

## Abstract (tr)

Hidrops fetalis, etkilenen fetüste kötü prognoza işaret eden ciddi bir durumdur. İzoimmün hidrops fetalis görülme sıklığı belirgin olarak azalmakla birlikte, giderek daha fazla sayıda nonimmün hidrops fetalis olgusu rapor edilmektedir. Hemoglobinopatiler nonimmün aracılı hidropsa neden olabilir. Bu makalede, homozigot α-talasemiye bağlı gelişen bir nonimmün hidrops fetalis olgusu sunuldu. Ülkemizde insidansın yüksek olması nedeniyle nonimmünhidrops fetalisli tüm olgularda α-talasemi araştırılmalıdır.

## INTRODUCTION

Hydrops fetalis is a serious condition that indicates poor prognosis for the affected fetus [1]. Its etiologies include cardiovascular abnormalities, placental malformations/problems, hematologic problems, congenital infections, noncardiac congenital anomalies, chromosomal aberrations, genetic syndromes, and miscellaneous causes. Isoimmune-or nonimmune-mediated mechanisms play a role in hematologic problems. Today, the incidence of isoimmune hydrops fetalis has decreased markedly. However, nonimmune hydrops fetalis cases have been more frequently reported. Nonimmune-mediated hydrops can be caused by hemoglobinopathies [2]. In this report we present a case of nonimmune hydrops fetalis caused by homozygous α-thalassemia.

## CASE REPORT

A 26-year-old gravida 3, para 1, abortus 2 woman was referred to the hospital at the 20th week of gestation because of hydrops fetalis. The mother and the father were first cousins. Ultrasound scan confirmed the presence of fetal ascites, generalized skin edema, and thickened placenta. After that, both the mother and the father were suspected to be thalassemia carriers with hematological parameters. The mother was noted to be anemic with a hemoglobin level of 105 g/L, hematocrit of 35.1%, and red blood cell indices including a mean corpuscular volume (MCV) of 73.2 fL, mean corpuscular hemoglobin (MCH) of 22.7 pg, mean corpuscular hemoglobin concentration (MCHC) of 311 g/L, and red cell distribution width (RDW) of 14.4%. The red blood cell morphology was notable for microcytic hypochromia. No further hematologic evaluation was performed on the parents at this time. 

**Figure 1:**
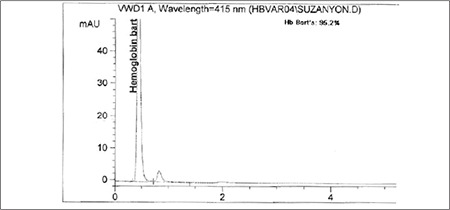
Hemoglobin electrophoresis of the patient showing high Hb Bart’s.

Although termination of pregnancy was offered, the couple decided to continue. During prenatal follow-up, no interventions, including fetal blood sampling or intrauterine transfusion, were performed. The woman went into preterm labor at 31 weeks of gestation. Emergency cesarean section was performed and a female baby weighing 1530 g was delivered. The placenta was large and edematous. The baby required immediate intubation due to respiratory distress. On initial exam, the abdomen was markedly distended secondary to hepatosplenomegaly and ascites. There was generalized skin edema. Hypoplastic thorax and bilateral pes varus were noted. Blood tests revealed a white blood count of 145 × 109/L, hemoglobin of 64 g/L, MCV of 97 fL, MCH of 25.5 pg, MCHC of 263 g/L, platelets of 276 × 109/L, reticulocyte count of 3%, total protein of 3.1 g/dL, and albumin of 1.6 g/dL (Table 1). There was no blood incompatibility with a negative direct antiglobulin test (Coombs test). The peripheral blood smear was notable for marked microcytosis, hypochromia, polychromasia, poikilocytosis, and anisocytosis with numerous erythroblasts. Hemoglobin electrophoresis revealed a high quantity of Hb Bart’s (95.2%) (Figure 1). Cytogenetic evaluation showed a normal karyotype of 46, XX. The patient died shortly after birth in spite of appropriate supportive treatment including oxygen, mechanical ventilation, fluid and electrolyte therapy, and blood transfusion.

After the patient’s death, the mother, the father, and a 9-year old sister had further evaluation for thalassemia. Hematological data of the patient and the family are shown in Table 1. The mother, the father, and the sister had low MCV and MCH, but high RBC and RDW. All had normal iron status. Mutation analysis using strip assay capable of detecting 22 mutations within the alpha genes showed that the father was heterozygous for a 20.5 kb double-gene deletion. No gene deletions were detected in the mother. It was recommended that the parents seek genetic counseling before becoming pregnant again.

## DISCUSSION

Alpha-thalassemia is a widespread genetic disorder throughout the world caused primarily by reduced synthesis of the α-globin chains, and it has been found at a high incidence in Turkey [3,4]. In 2007, Çürük et al. [3] reported the frequency of α-thalassemia to be 3% for the Çukurova region in the south of Turkey. Recently, in a single-center study, Guvenc et al. [4] evaluated 3000 individuals comprising premarital couples or patients with anemia living in Adana, a city in the Çukurova region. The authors found that the prevalence of α-thalassemia was higher (7.5%) in that region. 

The α-globin gene is located on chromosome 16p13.3 and mutations or deletions affecting either one or more α-globin genes cause α-thalassemia syndromes. The most frequent mutations of α-globin genes are single-gene deletions, and the deletion or inactivation of only one α-globin gene usually results in mild hematological findings. The α-thalassemia trait is caused by the deletion or inactivation of 2 α-thalassemia globin genes (--/αα or -α/-α). This results in mild microcytic, hypochromic anemia with normal or altered Hb A2 levels. If 3 of 4 α-globin genes become inactive, the affected individual has only 1 functional α-globin gene presenting with Hb H disease, which is characterized by severe anemia. In the Mediterranean area, widespread mutations are the -α (3.7) and –α (4.2) single α-globin gene deletions and the --MED and –α (20.5) double-gene deletions. In a study by Guvenc et al. [4], the most commonly detected mutations were –α (3.7), -α (4.2), --MED , --20.5 , α (PA-2 α), ααα (anti-3.7), and α (PA-1α). Their results showed that the α-thalassemia mutations represented a great heterogeneity and that the α (3.7) deletion had the highest frequency in Adana. We could not perform genetic analysis on the patient. However, the father was heterozygous for a 20.5 kb double-gene deletion. No gene deletions were detected in the mother, although her hematological parameters were consistent with thalassemia. We thought that the mother might have an undetected rare mutation. The parents were advised to obtain genetic counseling before becoming pregnant again.

Hydrops fetalis resulting from Hb Bart’s disease and molecular characterization of Hb H disease in Turkey have been reported [5]; however, the prevalence and distribution of deletional alpha-thalassemia, which is responsible for Hb Bart’s disease, is not known. In homozygous α-thalassemia, deletion of both copies of each of the 2 α-globin genes on chromosome 16 occurs, and thus no α-globin is produced. By 8 weeks of gestation, a switch to fetal hemoglobin production (hemoglobin F α2/γ2) occurs. Since α-globin chains are absent, hemoglobin F cannot be synthesized and hemoglobin Bart’s becomes the dominant hemoglobin. This results in a progressive severe anemia and tissue hypoxia. There is severe ineffective erythropoiesis with marked extramedullary hematopoiesis. All of these factors result in massive organomegaly, severe albuminemia, and heart failure leading to gross body edema, growth failure, and intrauterine demise [6,7]. Our patient presented with hydrops fetalis characterized by anemia, cardiac insufficiency, hepatosplenomegaly, and generalized edema. She was diagnosed with homozygous α-thalassemia with a high Hb Bart’s level detected at birth.

Developmental abnormalities are commonly seen in hydrops fetalis. It is likely that fetal hypoxia disturbs organogenesis and fetal development. In one series, 17% of affected newborns were found to have congenital anomalies. They included hydrocephaly and microcephaly, as well as cardiopulmonary, skeletal, and genitourinary malformations.

Hypoplasia of lungs, thymus, adrenals, and kidneys has been observed [8]. Hypoplastic thorax and bilateral pes varus were observed in the reported patient.

There is an increased incidence of serious maternal morbidity in these pregnancies, such as preeclampsia, dystocia, postpartum hemorrhage due to large placenta, and the psychological burden of carrying a nonviable fetus to term. It was estimated that half of these women died from complications resulting from these pregnancies [9]. No complications associated with hydrops fetalis developed in the mother of our patient, except premature labor.

Fetuses with homozygous α-thalassemia usually die in utero. Even if born alive, the prognosis is extremely poor, as almost all of them die within a few hours of birth. Therefore, any attempt at treatment should be started in utero [8]. The most ideal approach would be in utero stem cell transplantation before fetal immunological maturation [10]. However, this modality of treatment is still in the experimental stage. The alternative method is repeated in utero transfusion to prevent the complications of anemia, followed by postnatal transfusion while waiting for a suitable donor for bone marrow transplantation [11,12]. Unfortunately, no interventions could be performed in utero in the presented patient. She died shortly after birth despite supportive treatment.

Alpha-thalassemia is usually inherited in an autosomal recessive manner. The couples who have an offspring with Hb Bart’s disease represent the at-risk couples who are carriers of deletional alpha-thalassemia. Therefore, each child has a 25% chance of having Hb Bart’s disease. Prenatal testing may be carried out for couples who are at high risk of having a fetus with Hb Bart’s syndrome, or for a pregnancy in which one parent is a known α-thalassemia carrier and it is unknown whether the other parent has the mutation. In practice, any person found to have low erythrocyte MCV without iron deficiency should be considered a carrier of either α- or β-thalassemia mutation. Hemoglobin electrophoresis and HbA2 levels are often used as part of the laboratory investigations to diagnose thalassemia carriers. If the HbA2 level is elevated, the individual is considered to be a carrier of β-thalassemia mutation. If the HbA2 level is normal or low, the person is considered to be a carrier of α-thalassemia mutation. However, among people with microcytosis and high HbA2 levels, some are carriers of both α- and β-thalassemia mutations. Red cell indices of both parents and the sister showed microcytosis and hypochromia despite the lack of iron deficiency. HbA2 levels were higher than 3.5 in the father and the sister. Therefore, we thought that they might be carriers of both α-thalassemia and β-thalassemia. 

Recent studies have suggested that an immunocytological assay or a polymerase chain reaction-based DNA diagnostic technique can serve as alternative screening procedures. When parents have the α-thalassemia trait, DNA analysis of the fetus is required. Fetal tissue obtained by chorionic villus sampling early in the first trimester is indicated. Prenatal diagnosis for hydrops fetalis can also be conducted using fetal blood obtained by cordocentesis or amniocentesis [13]. From Turkey, Gurgey et al. were the first to report α-thalassemia-related hydrops fetalis in a fetus [14].

In our case, the parents were unaware of their carrier status for thalassemia until hydrops fetalis was diagnosed at the 20^th^ week of gestation. It was too difficult for the parents or the obstetrician to decide on termination of the pregnancy at that time. The mother’s hematological values were consistent with those of an α-thalassemia carrier. The patient’s hemoglobin electrophoresis revealed a high quantity of Hb Bart’s (95.2%). We want to emphasize that with carrier detection, timely genetic counseling, and the availability of prenatal diagnosis during early pregnancy, many couples at risk will be spared serious medical and psychological difficulties. We also indicate the importance of considering α-thalassemia in couples for whom red cell indices show microcytosis and hypochromia despite a lack of iron deficiency.

Because of the high incidence of the disease in our country [15,16], α-thalassemia should be investigated in all cases with nonimmune hydrops fetalis. 

Written consent was received from the parents.

**Conflict of Interest Statement**

The authors of this paper have no conflicts of interest, including specific financial interests, relationships, and/ or affiliations relevant to the subject matter or materials included.

## Figures and Tables

**Table 1:**
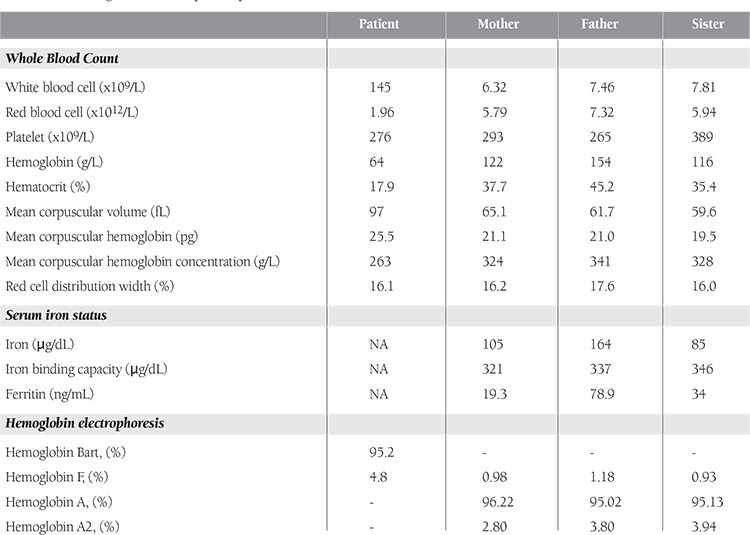
Hematological data of the patient, parents, and sister.
